# Molecular prevalence and socioeconomic, demographic, and behavioral risk factors of *Chlamydia trachomatis, Neisseria gonorrhoeae*, and *Ureaplasma* species among women in Mogadishu, Somalia

**DOI:** 10.1016/j.ijregi.2026.100931

**Published:** 2026-06-06

**Authors:** Jamal Hassan Mohamoud, Mohamed Hussein Adam, Ahmed Mahad Sheikh Mohamed, Bashiru Garba, Abdirahman Khalif Mohamud, Hassan Abdullahi Dahie, Hodo Aideed Asowe, Fartun Abdullahi Hassan Orey, Maryan Abdullahi Sh. Nur, Maryam Adawe, Jihaan Hassan, Najib Isse Dirie, Mohamed Mustaf Ahmed, Qadija Mohamud Ali, Mariam Hassan

**Affiliations:** a Department of Public Health, Faculty of Medicine and Health Sciences, SIMAD University, Mogadishu, Somalia.; b Department of Nursing and Midwifery, Faculty of Medicine and Health Sciences, SIMAD University, Mogadishu, Somalia.; c Department of Pediatrics and Child Health, Dr. Sumait Hospital, Faculty of Medicine and Health Sciences, SIMAD University, Mogadishu, Somalia; d Faculty of Medicine and Health Sciences, SIMAD University, Mogadishu, Somalia; e SIMAD Institute for Global Health SIMAD University, Mogadishu, Somalia

**Keywords:** Sexually transmitted infections, *Ureaplasma*, *Neisseria gonorrhoeae*, *Chlamydia trachomatis*, Women’s health, Multiplex real-time Polymerase Chain Reaction (PCR)

## Abstract

**Objectives:**

Sexually transmitted infections (STIs) are a major global public health concern with significant impacts on sexual and reproductive health. Data on STI prevalence and its associated risk factors in Somalia are limited.

**Methods:**

This hospital-based cross-sectional study was conducted from January to April 2023 at Dr. Sumait Hospital in Mogadishu. Genital swab samples were collected from 189 consenting women and analyzed using multiplex real-time polymerase chain reaction (PCR). The participants completed questionnaires on sociodemographic characteristics and behaviors. Logistic regression was used to identify factors associated with STI positivity.

**Results:**

The overall prevalence of at least one STI was 37.0% (95% CI: 30.2-43.9%). *Ureaplasma* was the most common (34.4%), followed by *Neisseria gonorrhoeae* (5.8%) and *Chlamydia trachomatis* (1.1%). In multivariable analysis, women aged 38-60 years had 2.8-fold higher odds of STI positivity compared to those aged 17-27 years (AOR=2.80, 95% CI: 1.124-7.002). Women with primary education had significantly higher odds of N. gonorrhoeae infection than those with postsecondary education.

**Conclusions:**

There is a high prevalence of STIs, particularly Ureaplasma, among women in Mogadishu. Older age and lower education levels were associated with increased STI risk. These findings highlight the need for enhanced STI screening and prevention efforts, especially among older and less-educated women in Somalia.

## Highlights

• Sexually transmitted infections prevalence among women in Mogadishu was 37.0%.
• *Ureaplasma*was the most frequently detected pathogen (34.4%).
• *Neisseria gonorrhoeae* and *Chlamydia trachomatis* were detected by polymerase chain reaction.
• Women aged 38-60 years had higher odds of sexually transmitted infections positivity.
• Lower education level was associated with gonorrhea infection.

## Introduction

Sexually transmitted infections (STIs) are a group of infectious diseases transmitted through unprotected sexual intercourse or intimate genital contact [1]. STIs are caused by more than 30 different bacterial, viral, and parasitic pathogens, among which the most common include *Treponema pallidum* (syphilis), *Neisseria gonorrhoeae, Chlamydia trachomatis, Trichomonas vaginalis*, hepatitis B virus, herpes simplex virus, human immunodeficiency virus (HIV), and human papillomavirus (HPV) [2]. This disease is of major global epidemiological and public health significance. Untreated STIs are associated with notable complications, including an increased risk of HIV and human papillomavirus transmission [3]. Recent reports by the World Health Organization show that over 1 million STIs are acquired every day worldwide, and more than 500 million individuals between the ages of 15 and 49 are reported to suffer from these infections [2]. Moreover, STIs have been shown to significantly affect the sexual and reproductive health of affected individuals due to stigmatization by members of society and spouses for married women/men, high rates of infertility and cancers, and pregnancy complications [4].

The symptoms of STIs can differ, ranging from slight suprapubic pain to dysuria, along with the presence of vaginal or urethral discharge and ulcerative sores. Unfortunately, most patients are asymptomatic, which often leads to serious complications, including permanent infertility, chronic pelvic pain, neonatal morbidity and mortality, and an increased risk of cervical cancer and HIV [5]. However, many STIs can be prevented by proper screening to identify both typical and atypical cases, particularly in individuals at high risk of infection [6]. Therefore, it has become paramount to estimate the prevalence of STIs to institute proper prevention and control measures and to provide adequate care for individuals infected with STIs. Co-infection with multiple STIs is common, especially among individuals with multiple sexual partners. Hence, a diagnostic approach that enables simultaneous detection of multiple target pathogens is essential for accurate diagnosis. In this regard, multiplex real-time polymerase chain reaction (PCR) has been found to be a highly sensitive method for detecting sexually transmitted pathogens [7]. This method is more advantageous because it is cost-effective, allows faster detection, and offers a shorter turnaround time than conventional methods, which are necessary for regular monitoring of treatment effectiveness once a patient has been diagnosed. In addition, understanding the molecular epidemiology of these diseases can offer valuable insights into genetic variability, propagation dynamics, and patterns of resistance to antimicrobial agents, which are emerging as serious public health challenges among STIs [4].

Somalia is among the poorest countries in the world, and with a rapidly growing population coupled with protracted armed conflicts and ongoing crises of drought and food insecurity, these have put a serious strain on the country’s already poor healthcare delivery services [8]. The instability and ongoing war against insurgency have led to the physical destruction of health facilities, compromised the medical supply chain, and displaced and migrated the qualified health workforce [9]. These situations are a recipe for the proliferation of STIs and increase the vulnerability of the population to these diseases [10]. There is a paucity of data on the burden of STDs in Somalia. Most reports were based on serological surveys conducted decades ago. Nonetheless, reports indicate that the incidence and prevalence rates of STIs are generally high in both urban and rural populations and vary considerably across areas, occupations, and sexes [[11], [12], [13]]. Moreover, in Somalia, routine screening for STIs is not a common practice because of its financial implications, thereby limiting the knowledge about transient carriers with the potential to continuously transfer pathogens. Unfortunately, there appears to be a shift in sexual behaviors, especially among youth with premarital sexual intercourse, and multiple sexual partners have become more common [14]. This study specifically investigated *Chlamydia trachomatis, Neisseria gonorrhoeae,* and *Ureaplasma* species because these pathogens are commonly associated with reproductive tract infections, infertility, pelvic inflammatory disease, and adverse pregnancy outcomes, yet remain underreported in Somalia. Therefore, this study was undertaken to estimate the prevalence of three STIs (chlamydia, gonorrhea, and Ureaplasma) among women seeking gynecological services in Mogadishu, Somalia. This would allow the determination of the occurrence of concomitant STIs, as well as the assessment of any association between the presence of STI pathogens and various socioeconomic and demographic risk factors among the study participants. This will be an important step towards efforts aimed at addressing the urgent need for surveillance of STIs and will provide a basis for improving outcomes.

## Methods and Materials

### *Study design and data collection*

A hospital-based cross-sectional study was conducted between January and April 2023 at the Dr. Sumait Hospital, affiliated with SIMAD University, Mogadishu, Somalia, to assess the prevalence and patterns of STIs among all consenting women of reproductive age who visited the hospital. After informing the participants of the study's objectives and protocol, consent was obtained before enrollment. All participants were required to complete a self-administered digital questionnaire to collect information related to their socioeconomic, demographic, and behavioral characteristics associated with acquiring STIs.

### *Sample size determination*

Based on previous studies, the reported proportion of the population with STIs was 13.3%. Therefore, the sample size was calculated as follows:

N=Z2×P(1−P)÷ε2)

where Z is 1.96 at a 95% confidence interval, e is the desired level of precision (5%), p is the reported prevalence (13.3%), and q is 1 - p.

Therefore

N=Z2×P(1−P)÷ε2

N=3.8416×0.133(1−0.133)÷0.0025

N=177.19samples

However, 189 samples were collected to increase the chances of detection.

### *Questionnaire data collection*

Trained nurses were responsible for administering the questionnaires to the participants. The questionnaire encompassed information about the socioeconomic, demographic, and behavioral profiles of the participants, including their medical and sexual history. Other important information, such as clinical symptoms including painful urination, pain during sexual intercourse, abdominal cramping, discharge, itching, bleeding in urine, and bleeding after sexual intercourse, was all recorded.

### *Genital sample collection and DNA extraction*

Genital swabs (endocervical and vaginal) were collected from each participant with the aid of a vaginal speculum and Cytobrush ® (DiaPath S.p.A, Italy), and the samples were placed in a sterile specimen bottle containing universal transport media and stored at -80°C prior to testing. DNA from 200 µl of each sample was extracted using a nucleic acid isolation kit (silica-based spin column) from Bioperfectus (Jiangsu Bioperfectus Technologies Co., Ltd., China) according to the manufacturer’s instructions. The final concentrated DNA was eluted in 50 µl of elution buffer.

### *Multiplex Real-time PCR for pathogen detection*

Real-time PCR was performed using the LineGene 9600 real-time PCR detection system (Bioer Technology, China). The multiplex real-time PCR kit used in this study was manufactured by Jiangsu Bioperfectus Technologies Co., Ltd., China (Catalog Number: PBP601). The assay simultaneously detects *Chlamydia trachomatis, Neisseria gonorrhoeae,* and *Ureaplasma* species through amplification of species-specific target gene regions according to the manufacturer’s instructions. Twenty microliters of PCR master mix containing primers and probes were pipetted into the reaction tube, 5 µL of the isolated DNA from the sample and controls (positive control and blank control) was added, and the tube was agitated using a vortex machine and briefly centrifuged for 30 s to ensure that all the reagents settled at the bottom, and then amplified in the thermocycler. The thermocycling conditions for multiplex qPCR were as follows: Uracil-DNA Glycosylase (UDG) treatment at 37°C for 5 min, pre-denaturation at 95°C for 10 min, followed by 40 cycles of denaturation at 95°C for 10 s, and annealing, extension, and fluorescent signal collection at 55°C for 40 s. PCR positivity was determined based on a cycle threshold (Ct) value of ≤38, while samples with Ct values >38 were considered negative.

## Statistical analysis

The results were analyzed using SPSS (version 20.0) and expressed descriptively in the form of frequencies and percentages appropriately to describe the studied population. The prevalence of STIs was calculated by dividing the number of participants who were positive for one or more STIs by the total sampled population. The association between exposures and outcomes was determined using the chi-square test, whereas logistic regression analysis was performed to determine the odds of infection.

### *Ethical consideration*

This study was approved by the ethical review board of the Institute of Medical Research, SIMAD University, Mogadishu, Somalia (Ref. no.: 2022/IMRSU/FMHS/FR18/P049). Verbal and written informed consent were obtained from all eligible women before they entered the study. The participants were informed about the objective of the study, that their participation was voluntary, and that they were free to withdraw at any moment without any repercussions.

## Results

### *Prevalence of sexually transmitted diseases*

This study reported that 37.0% of women tested positive for at least one STI, with a 95%CI of 30.2-43.9. The most frequently detected pathogen was *Ureaplasma* (34.4%), followed by Neisseria (5.8%) and Chlamydia (1.1%) (Table 1).

**Table 1:** Prevalence of Sexually Transmitted Infections among Study Participants.

Characteristics	Frequency (%)	95% Confidence Interval
**Sexually Transmitted Infections**		
Positive	70 (37.0)	30.2-43.9
Negative	119 (63.0)	56.1-69.8
**Ureaplasma**		
Positive	65 (34.4)	27.5-41.3
Negative	124 (65.6)	58.7-72.5
**Neisseria**		
Positive	11 (5.8)	2.6-9.5
Negative	178 (94.2)	90.5-97.4
**Chlamydia**		
Positive	2 (1.1)	-
Negative	187 (98.9)	-

### *Demographic characteristics*

Most of the respondents (49.2%) were in the age group between 28 and 38 years, with a mean age of 31.71(SD±8.905), and most of them (94.2%) were married. In addition, 75.1% were unemployed, 30.7% had informal education, and 64.6% earned a monthly income between USD 100 and 500 (Table 2). In the univariable logistic regression model, only two (2) variables were significantly associated with sexually transmitted diseases (STDs) at a significance level of 0.25. These variables were age and economic status, and were selected as candidates for inclusion in the multivariable logistic regression analysis. Variables with *P*-values less than 0.05 were considered statistically significant and are presented in Table 2. In multivariable logistic regression, those aged between 38 and 60 years had a 2.8-fold greater chance of becoming infected with at least one STI pathogen (AOR=2.80, 95%CI=1.124-7.002) than those aged between 17 and 27 years (Table 2).

**Table 2:** Sociodemographic characteristics and risk factors associated with STIs.

Characteristics	STI Status		OR (95% CI)	*P*-value	AOR (95% CI)	*P*-value
	Positive n (%)	Negative n (%)				
**Age group (In years)**						
17-27	29 (47.5)	32 (52.5)	1.00		1.00	
28-38	32 (34.4)	61 (65.6)	1.72 (0.893-3.342)	0.104	1.54 (0.786-3.055)	0.206
38-60	9 (25.7)	26 (74.3)	2.61 (1.054-6.500)	0.038	2.80 (1.124-7.002)	0.027
Mean = 31.71, Standard Deviation = 8.905						
**Marital Status**						
Married	65 (36.5)	113 (63.5)	0.69 (0.203-2.351)	0.553		
Divorced / Widowed	5 (45.5)	6 (54.5)	1.00			
**Education**						
Informal	21 (36.2)	37 (63.8)	1.00			
Primary School	22 (36.1)	39 (63.9)	1.00 (0.476-2.126)	0.987		
Secondary School	12 (42.9)	16 (57.1)	0.75 (0.301-1.900)	0.553		
Postsecondary	15 (35.7)	27 (64.3)	1.02 (0.446-2.338)	0.960		
**Employment Status**						
Employed	18 (38.3)	29 (61.7)	1.07 (0.544-2.121)	0.836		
Unemployed	52 (36.6)	90 (63.4%)	1.00			
**Economic Status**						
Less than 100 $	18 (41.9)	25 (58.1)	1.00			
100 $-500 $	47 (38.5)	75 (61.5)	1.14 (0.566-2.330)	0.700		
More than 500 $	5 (20.8)	19 (79.2)	2.73 (0.861-8.697)	0.088		
**Ever heard about STI?**						
No	50 (37.3)	84 (62.7)	0.96 (0.500-1.842)	0.902		
Yes	20 (36.4)	35 (63.6)	1.00			
**Dyspareunia awareness**						
No	44 (38.6)	70 (61.4)	0.84 (0.460-1.549)	0.584		
Yes	26 (34.7)	49 (65.3)	1.00			
**Knowledge of STI prevention**						
No	52 (39.4%)	80 (60.6)	0.71 (0.367-1.372)	0.308		
Yes	18 (31.6%)	39 (68.4)	1.00			
**Early sexual debut**						
No	53 (36.3)	93 (63.7)	1.14 (0.571-2.306)	0.700		
Yes	17 (39.5)	26 (60.5)	1.00			
**Contraceptive use**						
No	51 (36.7)	88 (63.3)	1.05 (0.543-2.061)	0.869		
Yes	19 (38.0)	31 (62)	1.00			
**Multiple partners**						
No	54 (39.1)	84 (60.9)	0.7 (0.359-1.408)	0.328		
Yes	16 (31.4)	35 (68.6)	1.00			
**Vaginal bleeding**						
No	57 (37.5)	95 (62.5)	0.90 (0.426-1.912)	0.789		
Yes	13 (35.1)	24 (64.9)	1.00			
**Vaginal discharge**						
No	57 (36.5)	99 (63.5)	1.12 (0.522-2.440)	0.758		
Yes	13 (39.4)	20 (60.6)	1.00			
**STI treatability**						
No	62 (37.6)	103 (62.4)	0.83 (0.336-2.054)	0.688		
Yes	8 (33.3)	16 (66.7)	1.00			
**Multiparity**						
No	66 (37.3)	111 (62.7)	0.84 (0.244-2.901)	0.784		
Yes	4 (33.3)	8 (66.7)	1.00			
**Smoking**						
No	63 (38.2)	102 (61.8)	0.66 (0.262-1.697)	0.395		
Yes	7 (29.2)	17 (70.8)	1.00			

STIs, sexually transmitted infections

### *Risk Factors associated with Ureaplasma Infection*

In the univariate logistic regression model, only two (2) variables were significantly associated with Ureaplasma at a significance level of 0.25. These variables were age and economic status, and were selected as candidates for inclusion in the multivariable logistic regression analysis. Variables with a *P*-value less than 0.05 were considered statistically significant. In multivariable logistic regression, women aged 38-60 years were 3.6 times more likely to become infected with Ureaplasma (95% CI=1.124-7.002) compared to women aged 17-27 years (Table 3).

**Table 3:** Sociodemographic characteristics and risk factors associated with Ureaplasma.

Characteristics	Ureaplasma		OR (95% CI)	*P*-value	AOR (95% CI)	*P*-value
	Positive n (%)	Negative n (%)				
**Age group (In years)**						
17-27	28 (45.9)	33 (54.1)	1.00		1.00	
28-38	30 (32.3)	63 (67.7)	1.78 (0.916-3.466)	0.089	1.60 (0.81-3.17)	0.176
38-60	7 (20.0)	28 (80.0)	3.39 (1.287-9.947)	0.013	3.60 (1.36-9.54)	0.010
Mean = 31.71, Standard Deviation = 8.905						
**Marital Status**						
Married	61 (34.3)	117 (65.7)	1.09 (0.309-3.891)	0.887		
Divorced / Widowed	4 (36.4)	7 (63.6)	1.00			
**Education**						
Informal	20 (34.5)	38 (65.5)	0.95 (0.410-2.199)	0.905		
Primary School	21 (34.4)	40 (65.6)	0.95 (0.415-2.186)	0.908		
Secondary School	10 (35.7)	18 (64.3)	0.90 (0.330-2.458)	0.837		
Postsecondary	14 (33.3)	28 (66.7)	1.00			
**Employment Status**						
Employed	15 (31.9)	32 (68.1)	1.00			
Unemployed	50 (35.2)	92 (64.8)	0.86 (0.427-1.743)	0.680		
**Economic Status**						
Less than 100 $	17 (39.5)	26 (60.5)	1.00			
100 $-500 $	43 (35.2)	79 (64.8)	0.40 (0.126-1.283)	0.124		
More than 500 $	5 (20.8)	19 (79.2)	0.48 (0.169-1.386)	0.176		
**Ever heard about STIs?**						
No	45 (33.6)	89 (66.4)	1.13 (0.586-2.178)	0.715		
Yes	20 (36.4)	35 (63.6)	1.00			
**Dyspareunia awareness**						
No	42 (36.8)	72 (63.2)	0.75 (0.407-1.411)	0.382		
Yes	23 (30.7)	52 (69.3)	1.00			
**Knowledge of STIs prevention**						
No	48 (36.4)	84 (63.6)	0.74 (0.381-1.452)	0.386		
Yes	17 (29.8)	40 (70.2)	1.00			
**Early sexual debut**						
No	50 (34.2)	96 (65.8)	1.02 (0.504-2.201)	0.938		
Yes	15 (34.9)	28 (65.1)	1.00			
**Contraceptive use**						
No	47 (33.8)	92 (66.2)	1.10 (0.560-2.165)	0.780		
Yes	18 (36.0)	32 (64.0)	1.00			
**Multiple partners**						
No	50 (36.2)	88 (63.8)	0.73 (0.366-1.470)	0.382		
Yes	15 (29.4)	36 (70.6)	1.00			
**Vaginal bleeding**						
No	52 (34.2)	100 (65.8)	1.04 (0.490-2.213)	0.915		
Yes	13 (35.1)	24 (64.9)	1.00			
**Vaginal discharge**						
No	53 (34.0)	103 (66.0)	1.11 (0.508-2.429)	0.793		
Yes	12 (36.4)	21 (63.6)	1.00			
**STIs treatability**						
No	57 (34.5)	108 (65.5)	0.94 (0.382-2.347)	0.907		
Yes	8 (33.3)	16 (66.7)	1.00			
**Multiparity**						
No	61 (34.5)	116 (65.5)	0.95 (0.275-3.284)	0.936		
Yes	4 (33.3)	8 (66.7)				
**Smoking**						
No	58 (35.2)	107 (64.8)	0.76 (0.298-1.938)	0.565		
Yes	7 (29.2)	17 (70.8)	1.00			

STIs, sexually transmitted infections

### *Risk Factors associated with Neisseria Infection*

In the univariate logistic regression model, only three (3) variables were significantly associated with Neisseria infection at a significance level of 0.25. These variables were education, ever heard of STI, and long-term use of contraceptives. These variables were selected as candidates for inclusion in the multivariable logistic regression, and variables with a *P*-value less than 0.05 were considered statistically significant. In multivariable logistic regression, women with a primary education level were 10 times more likely (95%CI=1.060-101.844) to become infected with Neisseria compared to those with postsecondary education (Table 4).

**Table 4:** Sociodemographic characteristics and risk factors associated with Neisseria.

Characteristics	Neisseria		OR (95% CI)	*P*-value	AOR (95% CL)	*P*-value
	Positive n (%)	Negative n (%)				
**Age group (In years)**						
17-27	5 (8.2)	56 (91.8)	0.67 (0.125-3.698)	0.654		
28-38	4 (4.3)	89 (95.7)	1.34 (0.236-7.712)	0.737		
38-60	2 (5.7)	33 (94.3)	1.00			
Mean = 31.71, Standard Deviation = 8.905						
**Marital Status**						
Married	10 (5.6)	168 (94.4)	0.69 (0.203-14.457)	0.637		
Divorced / Widowed	1 (9.1)	10 (90.9)	1.00			
**Education**						
Informal	3 (5.2)	55 (94.8)	1.93 (0.408-9.120)	0.407	3.62 (0.704-18.70)	0.134
Primary School	1 (1.6)	60 (29.1)	6.31 (0.680-58.658)	0.105	10.38 (1.06-16.84)	0.044
Secondary School	3 (10.7)	25 (89.3)	0.87 (0.181-4.258)	0.871	1.24 (0.23-6.50)	0.799
Postsecondary	4 (9.5)	38 (90.5)	1.00		1.00	
**Employment Status**						
Employed	4 (8.5)	43 (91.5)	1.00			
Unemployed	7 (4.9)	135 (95.1)	1.79 (0.501-6.424)	0.369		
**Economic Status**						
Less than 100 $	3 (7.0)	40 (93)	1.00			
100 $-500 $	8 (6.6)	114 (93.4)	1.06 (0.270-4.227)	0.924		
More than 500 $	0	24 (100)		0.998		
**Ever heard about STI**						
No	10 (7.5)	124 (92.5)	0.23 (0.02-1.83)	0.166		
Yes	1 (1.8)	54 (98.2)	1.00			
**Dyspareunia awareness**						
No	8 (7.0)	106 (93.0)	0.55 (0.142-2.152)	0.392		
Yes	3 (4.0)	72 (96.0)	1.00			
**Knowledge of STI prevention**						
No	7 (5.3)	125 (94.7)	1.34 (0.379- 4.798)	0.645		
Yes	4 (7.0)	53 (93.0)	1.00			
**Early sexual debut**						
No	8 (5.5)	138 (94.2)	1.29 (0.328-5.106)	0.713		
Yes	3 (7.0)	40 (93.0)	1.00			
**Contraceptive use**						
No	10 (7.2)	129 (92.8)	0.26 (0.033-2.111)	0.209		
Yes	1 (2.0)	49 (98.0)	1.00			
**Multiple partners**						
No	9 (6.5)	129 (93.5)	0.58 (0.122-2.804)	0.503		
Yes	2 (3.9)	49 (96.1)	1.00			
**Vaginal bleeding**						
No	8 (5.3)	144 (94.7)	1.58 (0.400-6.304)	0.511		
Yes	3 (8.1)	34 (91.9)	1.00			
**Vaginal discharge**						
No	10 (6.4)	146 (93.6)	0.45 (0.056-3.692)	0.462		
Yes	1 (3.0)	32 (97.0)	1.00			
**STI treatability**						
No	10 (6.1)	155 (93.9)	0.67 (0.082-5.513)	0.713		
Yes	1 (4.2)	23 (95.8)	1.00			
**Multiparity**						
No	11 (6.2)	166 (93.8)	000	0.999		
Yes	0	12 (100)	1.00			
**Smoking**						
No	10 (6.1)	155 (93.9)	0.67 (0.082-5.513)	0.713		
Yes	1 (4.2)	23 (95.8)	1.00			

STIs, sexually transmitted infections

## Discussion

This investigation was conducted to simultaneously detect important sexually transmitted infectious disease agents among women using a real-time PCR diagnostic test known for its reliability, sensitivity, and cost-effectiveness. According to the WHO, the diagnosis of STIs using molecular technology, as in this study, is especially useful for the diagnosis of asymptomatic infections. However, it is not very common in most low- and middle-income countries. The findings from this study showed that 37% of the women harbored at least one STI pathogen, with Ureaplasma (34.4%) having the highest number of positive detections, followed by Neisseria (5.8%) and Chlamydia (1.1%). Literature on the status of STIs among the general Somali population is scarce, with the most available publications following Internet searches being an integrated HIV and STIs bio-behavioral survey (IBBS) conducted among key populations in Somalia, including female sex workers, truckers, uniformed personnel, and port workers. The study reported varying levels of infection, with HIV prevalence ranging from 0.2-2.9% and syphilis with 1.2-6.3%, and female sex workers were found to have the highest prevalence [15]. The other two publications were sexually transmitted viral infections in various populations, as well as STIs among men in Mogadishu, Somalia. For each of these studies, the prevalence of hepatitis B, cytomegalovirus infection, and herpes simplex viral infection was all found to be high, with no participant reporting positivity for HIV [12,16]. These results indicate that STIs are high in Mogadishu, which corresponds to the results of the present study. Globally, the burden of STIs is unevenly distributed, with resource-limited countries in Africa among the countries with the highest burden, particularly those affected by conflict, where healthcare services have been disrupted [17,18]. The findings of this study are comparable to reports from other low-income and conflict-affected countries in sub-Saharan Africa, where limited access to STI screening, poor healthcare infrastructure, and low public awareness contribute to a persistently high burden of sexually transmitted infections.

In this study of 189 sexually active Somali women, *Ureaplasma urealyticum* was the most detected pathogen, with a prevalence of 34.4%. Although *U. urealyticum* is a commensal of the human urogenital tract, it can cause a variety of diseases and events, including non-gonococcal urethritis, bacterial vaginosis, chorioamnionitis, and pelvic inflammatory diseases [[19], [20], [21]]. Similarly, bacteria are frequently isolated from spontaneously aborted fetuses, stillborn fetuses, and premature infants [21,22]. Unfortunately, data regarding the relative frequencies of ureaplasmas in the Somali population are nonexistent. Although *Ureaplasma* species are frequently considered commensal organisms colonizing the lower genital tract, increasing evidence suggests that they may become pathogenic under certain conditions, particularly among immunocompromised individuals and pregnant women. Their detection in this study may therefore represent either colonization or active infection. However, the high prevalence observed highlights the need for further clinical and microbiological investigations to determine their pathogenic role among Somali women. *Ureaplasmas* are generally considered low-virulence pathogens that inhabit the lower genitourinary tract of females. However, the organism has demonstrated enhanced virulence, especially among immunosuppressed pregnant women, and entry into the amniotic fluid is usually followed by inflammatory reactions with an increased risk of preterm labor and neonatal morbidity [23].

Other important sexually transmitted pathogens detected in this study were *Neisseria gonorrhea* and *Chlamydia trachomatis*, with a prevalence of 5.8% and 1.1%, respectively. The contributions of *N. gonorrhoeae* and *C. trachomatis* to the disease burden in Somalia remain largely unknown. Although the prevalence of *C. trachomatis* was very low, the prevalence of *N. gonorrhoeae* was within the range estimated for the sub-Saharan African region (2-15%) [24,25]. Gonorrhea is a common, STI that usually spreads through unprotected sexual intercourse. Although it is a treatable and curable disease, when left untreated, mostly due to its asymptomatic clinical manifestation, it can lead to pelvic inflammatory disease, infertility, and other pregnancy-related problems among women, while common symptoms in men include painful urination and discharges [26,27]. Prior to this study, the status of *N. gonorrhea* in the general population of Somalia was largely unknown. Two studies were conducted in a Somali village in 1990, where no positive isolation was recorded, and another study reported an epidemic of *N. gonorrhea* in a Somali orphanage, where a 56% prevalence was reported among 95 girls aged 6-14 years [28,29].

The high prevalence observed in the Somali orphanage study may be because the study population is vulnerable and is among the high-risk groups for gonorrhea. Second, the high detection rate may be due to cross-reactivity and false-positive detection, which are common limitations of serological techniques [30,31]. The prevalence of *N. gonorrhea* recorded in this study was also lower than the 20.8% reported in neighboring Ethiopia among patients visiting Gondar Hospital. These discrepancies might be due to the diagnostic technique used, which in our case was real-time PCR, which is highly sensitive, unlike serological techniques that are fraught with cross-reactivity and false-positive results. This study also examined the relationship between STIs and the demographic characteristics of the participants. A wide range of social, economic, and cultural factors can contribute to the spread of STDs. These include multiple sexual partners, unprotected sexual encounters, age, and gender [32]. Despite the variable association observed with patient demographics, only age and education were found to be statistically significant. Participants aged 38-60 were found to have twice as much risk of infection as those aged 17-27-year-olds. This finding is consistent with that of a 6-year retrospective study conducted in a tertiary care hospital in New Delhi, which revealed an increasing trend in the number of patients over 60 years of age diagnosed with STIs [33]. Recently, concerns have been raised regarding the incidence of HIV and other STIs among aged individuals, which is said to be increasing in some regions of the world. This is mainly attributed to the fact that, as we age, our health typically decreases, and our immune system becomes weaker and more susceptible to infections, including STIs such as syphilis, chlamydia, and gonorrhea. Hence, it has become necessary to destigmatize STDs among the elderly and encourage their inclusion in screening programs, as opposed to the current practice where attention is given more to adolescents and youths. On the other hand, our assessment of the association between educational level and the risk of STI showed that individuals with low educational attainment have a significantly higher risk of contracting STIs. This finding is in agreement with many other studies that have assessed the effect of educational level on STIs. For instance, a study undertaken to determine the risk of sexually transmitted infections among clients of Dutch sexual health centers revealed that lower educational levels were associated with gonorrhea in women [34]. In a related study among commercial sex workers in Quito, Ecuador, lower educational levels were associated with more STIs [35]. The consistently high rate of STIs among those with low knowledge highlights the need for educational interventions in the study population.

## Limitations

This study had several limitations. First, it was a single-center hospital-based study, which may limit the generalizability of the findings to the wider Somali population. Second, the relatively small sample size may have reduced the statistical power to detect significant associations for some behavioral risk factors. Third, some expected risk factors, such as multiple sexual partners, contraceptive use, and unprotected sexual intercourse, were not statistically significant, possibly because of underreporting of sensitive sexual behaviors due to stigma and cultural barriers. Lastly, only three bacterial sexually transmitted pathogens were investigated, while viral and parasitic STIs were not included.

## Conclusion

This study demonstrated a high prevalence of STIs among women in Mogadishu, Somalia, with *Ureaplasma* species being the most commonly detected pathogen, followed by *Neisseria gonorrhoeae* and *Chlamydia trachomatis*. Older age and lower educational level were significantly associated with increased risk of infection. The findings highlight the importance of molecular diagnostic approaches such as multiplex real-time PCR for accurate detection of symptomatic and asymptomatic STIs in resource-limited settings. These findings provide important baseline epidemiological data for Somalia and emphasize the need to strengthen STI prevention and control strategies. Community awareness programs, routine screening services, sexual health education, and improved access to reproductive healthcare should be prioritized. In addition, integrating STI surveillance and screening into primary healthcare services may improve early detection, timely treatment, and the reduction of STI-related complications among Somali women.

## CRediT authorship contribution statement

**Jamal Hassan Mohamoud:** Conceptualization, Writing – original draft. **Mohamed Hussein Adam:** Methodology, Validation, Writing – original draft. **Ahmed Mahad Sheikh Mohamed:** Formal analysis, Writing – original draft. **Bashiru Garba:** Formal analysis, Methodology, Writing – original draft. **Abdirahman Khalif Mohamud:** Formal analysis. **Hassan Abdullahi Dahie:** Formal analysis, Supervision. **Hodo Aideed Asowe:** Supervision, Validation. **Fartun Abdullahi Hassan Orey:** Formal analysis, Supervision, Writing – original draft, Writing – review & editing. **Maryan Abdullahi Sh. Nur:** Investigation, Methodology, Supervision. **Maryam Adawe:** Formal analysis, Supervision. **Jihaan Hassan:** Formal analysis, Writing – original draft. **Najib Isse Dirie:** Investigation, Writing – original draft. **Mohamed Mustaf Ahmed:** Writing – original draft. **Qadija Mohamud Ali:** Methodology, Validation. **Mariam Hassan:** Methodology, Writing – original draft.

## Declaration of competing interest

The authors have no competing interests to declare.
